# A commentary on “Thoracoscopic surgical ablation for atrial fibrillation patients with functional regurgitation: the treatment strategy prioritizing atrial fibrillation”

**DOI:** 10.1097/JS9.0000000000002973

**Published:** 2025-07-07

**Authors:** Xuesi Chen, Yingchun Bao, Qijun Zhang

**Affiliations:** Department of Cardiology, The Affiliated People’s Hospital of Ningbo University, Ningbo, Zhejiang, China

*Dear Editor,*

We read with great interest the recent article by Sun and colleagues entitled “Thoracoscopic surgical ablation for atrial fibrillation patients with functional regurgitation: the treatment strategy prioritizing atrial fibrillation”[1]. The authors present important findings regarding the efficacy of thoracoscopic surgical ablation (TSA) in managing atrial fibrillation (AF) and its associated functional regurgitation (FR), particularly functional mitral regurgitation. While their work contributes meaningfully to this evolving field, several key aspects merit further discussion. This correspondence is compliant with the TITAN Guidelines 2025—governing declaration and use of AI[2].

Firstly, the rationale for prioritizing rhythm control in the management of atrial fibrillation warrants deeper mechanistic clarification. AF is a primary driver of progressive atrial remodeling[3], leading to annular dilatation and FR, specifically atrial functional mitral regurgitation and atrial functional tricuspid regurgitation. The latter, which is closely linked to AF, is associated with poorer clinical outcomes, including increased mortality and higher rates of major adverse cardiovascular events[4]. While successful rhythm control can induce reverse remodeling, this study could benefit from a more explicit discussion of how AF ablation is expected to reduce FR severity and the specific mechanisms involved. Furthermore, quantifying the FR improvement post-ablation and correlating this with patient-centered outcomes would strengthen the clinical relevance of the findings.

Secondly, the accuracy of reported AF recurrence rates depends heavily on the monitoring methodology. Short-term, intermittent monitoring, such as 24–48 hour Holter ECGs, significantly underestimates recurrence, especially in cases of paroxysmal or asymptomatic episodes. Implantable loop recorders offer superior diagnostic fidelity and should be considered in future study protocols[5]. Additionally, incorporating objective indicators of cardiac reverse remodeling, such as left atrial reverse remodeling, would offer a more comprehensive and prognostically informative assessment of therapeutic success.

Lastly, future research should focus on personalized strategies. Current patient selection based on AF type alone is often inaccurate. A more personalized approach, integrating advanced imaging, electrophysiological parameters, and machine learning models, may enhance patient selection and tailor ablation strategies[6]. Future multicentre randomized controlled trials with extended follow-up and continuous monitoring will be essential to validate TSA against alternative strategies and to clarify its long-term impact on clinical outcomes.

In conclusion, Sun *et al*’s study provides valuable insight into the role of TSA in managing AF patients with FR[1]. Addressing the outlined issues will advance the field toward more precise and effective therapeutic approaches tailored to the individual patient.

## Data Availability

All data are included in the article.
